# Patch Infection Following Carotid Endarterectomy: A Single-Center Audit and Literature Review

**DOI:** 10.7759/cureus.65420

**Published:** 2024-07-26

**Authors:** Konstantinos Roditis, Sofia Tzamtzidou, Konstantinos Maltezos, Afroditi Antoniou, Nikolaos Giannakopoulos, Paraskevi Tsiantoula, Theofanis Papas

**Affiliations:** 1 Department of Vascular Surgery, Korgialenio-Benakio Hellenic Red Cross Hospital, Athens, GRC; 2 Department of Vascular Surgery, KAT Attica General Hospital, Athens, GRC

**Keywords:** post operative complication, postoperative wound infection, carotid artery disease, carotid artery surgery, synthetic patch, audit, cea, carotid pseudoaneurysm, patch infection, carotid endarterectomy

## Abstract

Introduction: Carotid endarterectomy (CEA) is a surgical procedure that carries a rare but serious risk of patch infection. This study examines the management and outcomes of patch infections in CEA patients treated in our department over 23 years. A literature review of studies on prosthetic patch infection following CEA published from January 1992 up to December 31, 2022 was also carried out.

Methods: We conducted a retrospective audit of patients who underwent CEA in a hospital in Athens, Greece, between January 1, 1999, and December 31, 2022.

Results: Between January 1999 and December 2022, we treated seven patients with carotid patch infections who had their original CEA at our department. *Staphylococcus* *epidermidis* and *Staphylococcus aureus* were the most common infecting organisms. One patient (14%) died from hemorrhagic shock before surgery, while the remaining six (86%) underwent debridement, patch excision, and great saphenous vein patching. No peri-operative deaths or strokes occurred, and there were no re-infections during a median follow-up of 159 months.

Conclusions: Excision of infected material followed by revascularization using a vein graft remains the prevailing treatment.

## Introduction

Carotid arterial endarterectomy and reconstruction can be performed by a range of techniques including standard endarterectomy and primary closure, eversion endarterectomy and re-implantation of the internal carotid artery (ICA), and endarterectomy with patch angioplasty [1]. The patch used for arterial reconstruction can be autologous, using the great saphenous vein, the anterior facial vein, the internal jugular vein, or some other vein graft. Woven polyester (Dacron) or expanded polytetrafluoroethylene (ePTFE) patches have been widely used, while some surgeons use bovine pericardium-derived patches [2]. The wide use of synthetic grafts is due to the fact that they have the same results compared to venous patches with regard to recurrent re-stenosis and peri-operative stroke rate [3]. Nevertheless, infection of the synthetic patch is a rare but serious complication of carotid endarterectomy. We present seven cases of synthetic patch infection, as well as a literature review of relevant publications focusing on patch infection (diagnosis, bacterial flora, and management).

## Materials and methods

Study design

This was a retrospective audit conducted at the Department of Vascular Surgery at Korgialenio-Benakio Hellenic Red Cross General Hospital, Athens, Greece, of patients with prosthetic patch infection following CEA. Our institutional scientific/audit board confirmed that our study did not require an ethical permit according to national law and regulations, since it was merely an evaluation of performance. Patients in whom patch infection was suspected and who then presented or were referred to our department for treatment were included in the study.

Between January 1, 1999, and December 31, 2022, 920 patients underwent CEA in the Department of Vascular Surgery at Korgialenio-Benakio Hellenic Red Cross General Hospital. Of these, 253 (27.5%) underwent reversed endarterectomy, 115 (12.5%) common carotid (CCA)-internal carotid artery (ICA) ePTFE bypass and 46 (5%) bovine pericardium patch closure, and were excluded from the study, leaving 506 (55%) patients who underwent prosthetic patch closure (483 polyester, 23 ePTFE).

Initial operation

All 506 patients underwent carotid endarterectomy (CEA) with general anesthesia. The procedure included systemic heparinization (5000 IU of unfractionated heparin), intravenous prophylactic antibiotics, standard tacking, and routine prosthetic patching. Antibiotic prophylaxis was provided with three doses of intravenous cefuroxime (1 g). The first dose was administered immediately before the induction of anesthesia, followed by additional doses every eight hours thereafter.

Post-operative course after index CEA

Patients were monitored in the post-anesthetic room for one to two hours. Blood pressure was strictly monitored and maintained under 140/90 mmHg by administering intravenous sodium nitroprusside. All patients were discharged on postoperative day 2. Patients were followed up in the outpatient clinic at two weeks and thereafter in three, six, and 12 months, and given instructions to return every year for re-evaluation or if any new symptoms or wound complications developed.

Carotid patch infection management

All patients with suspected patch infection had routine blood tests (hematology/biochemistry) and blood cultures taken, as well as microbiological swabs from any discharging wounds. All patients were started on parenteral antibiotics (meropenem 2 g every eight hours and vancomycin 1 g every 12 hours), revised once cultures/sensitivities and therapeutic vancomycin serum levels were available, and continued for two weeks following surgery or until there was no clinical evidence of infection.

A detailed color duplex ultrasound (unless contraindicated by massive hemorrhage) was first done by an experienced vascular ultrasonographist in order to obtain detailed information about the extent of surrounding tissue infection, presence or absence of abscess or serous infiltration, exclude the presence of a false aneurysm, and to establish the patency of the ICA, external carotid artery (ECA), and CCA, as well as patch corrugation, which has been recognized as a warning sign of patch infection [4]. In addition, a digital subtraction arteriogram (DSA) was performed in order to further evaluate the presence of a pseudoaneurysm and/or the accessibility of the distal part of the ICA and to guide us in designing our open surgical approach. No cross-sectional imaging (CT angiography (CTA)/MRI) was performed in any of the patients, as this was not considered necessary, since a detailed diagnostic evaluation had been provided already by color duplex ultrasound workup. Furthermore, at the time our patients were treated (the last patient treated in 2012), a DSA was much more easily done in a timely manner at our department (being located in a major trauma center) than a CTA, due to the high volume of patients needing a CT-scan at the emergency department. Also, due to the high volume of diagnostic DSA being done at our department at that time, complication rates (given its invasive nature as a diagnostic modality) were quite low (<1%). No DSA was performed intraoperatively in any of the cases.

The surgical approach remained consistent in the majority of cases. The initial step involved gaining control over the proximal CCA. Following this, the original incision was carefully reopened, ensuring the preservation of the hypoglossal and vagus nerves, while exposing the distal ICA. Whenever feasible, arterial reconstruction (utilizing saphenous venous patches) was carried out following the thorough removal of macroscopically infected tissues and patch removal. Subsequently, and since no multi-resistant pathogens were isolated from initial wound cultures, while all cases had demonstrated only mild clinical and laboratory signs of infection, all incisions were closed and 12-Fr wound drains were inserted.

## Results

**Figure 1:**
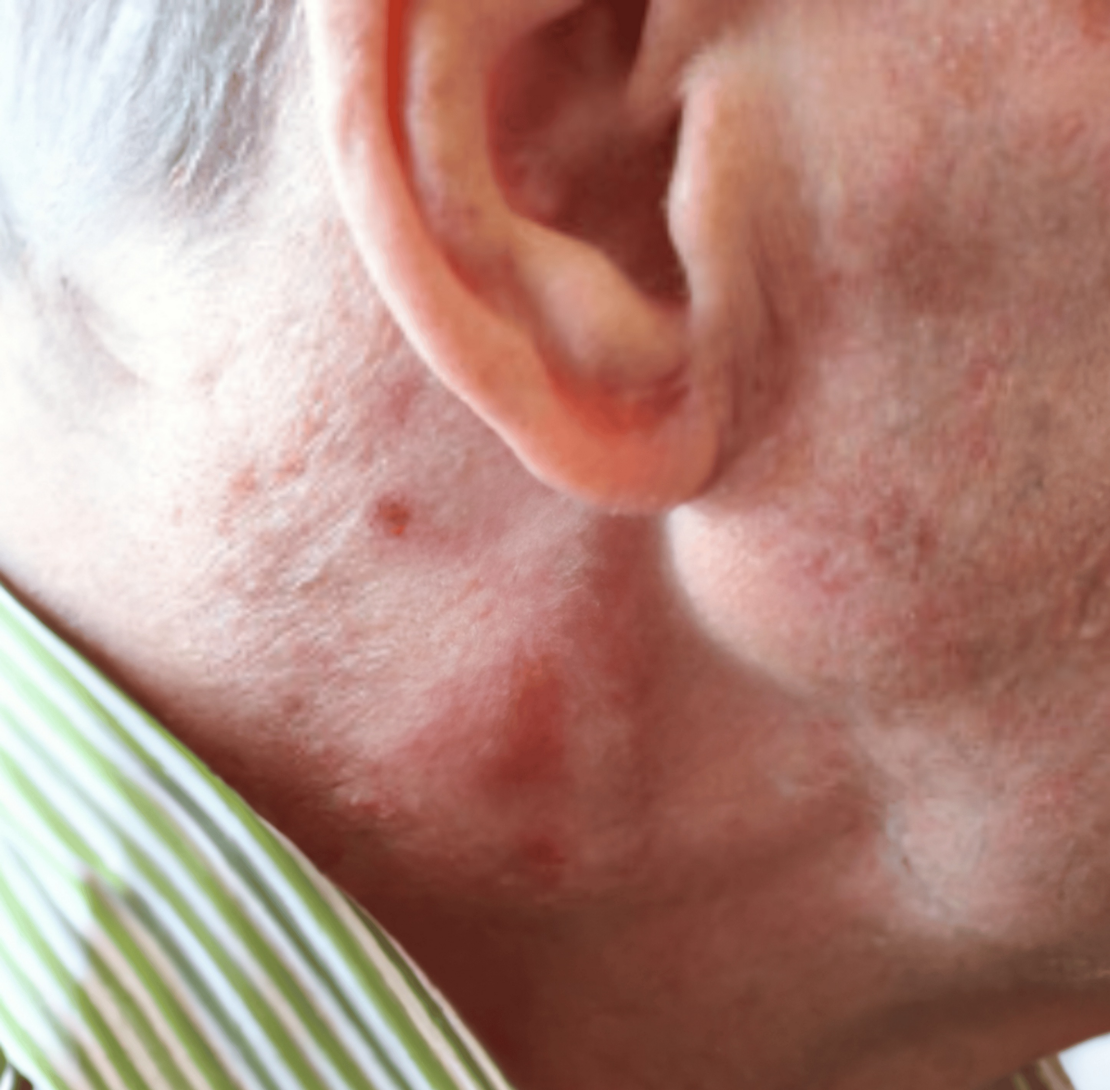
Draining sinus and neck swelling adjacent to CEA surgical wound CE: carotid endarterectomy

**Figure 2:**
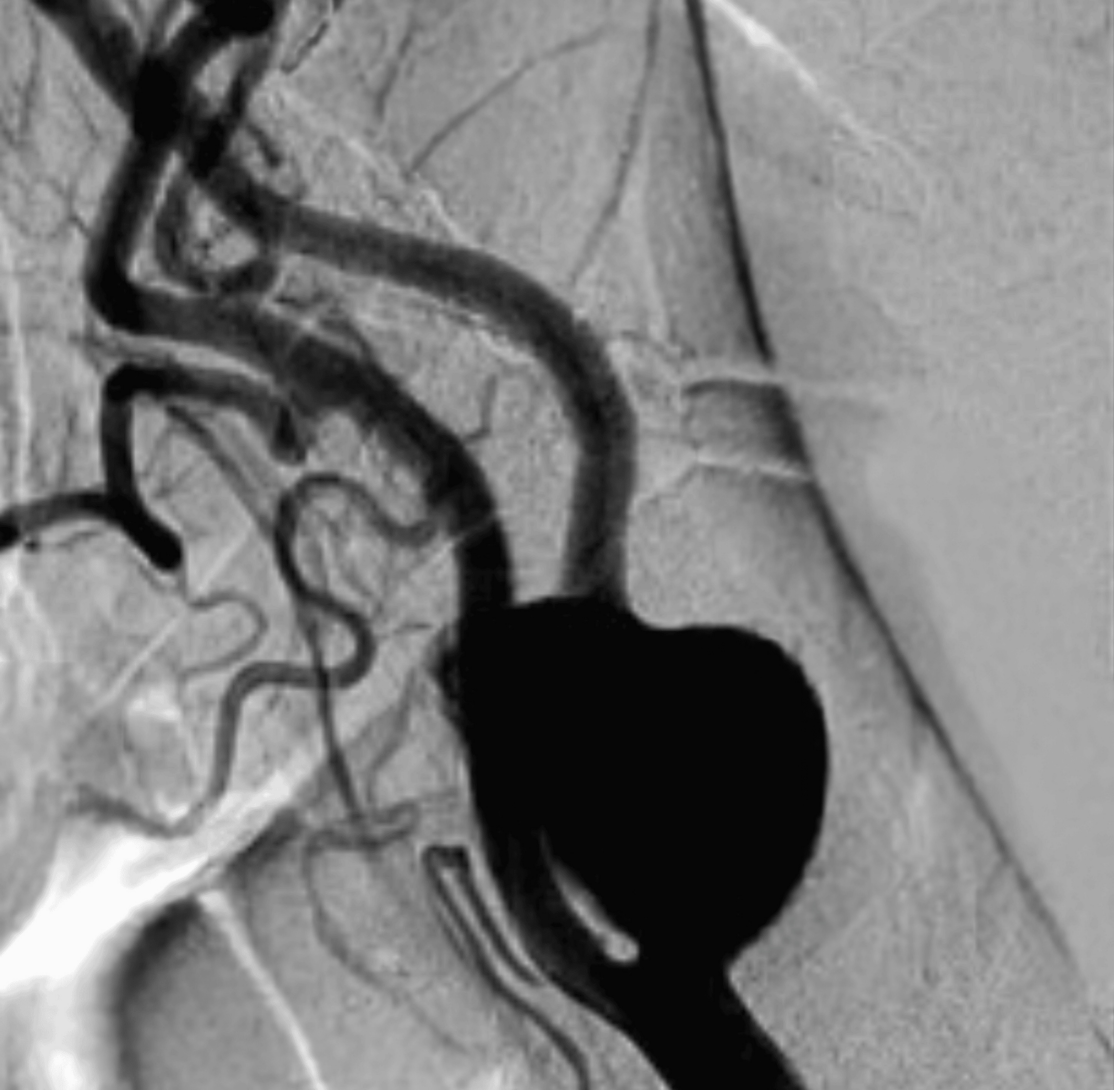
DSA showing an ICA pseudoaneurysm formation after CEA DSA: digital subtraction angiogram; ICA: internal carotid artery; CEA: carotid endarterectomy

Between January 1999 and December 2022, 506 patients were treated in our center with synthetic patch-CEA. Of these, seven (1.38%) were subsequently treated for synthetic patch infection. The average age of those patients was 69 years (range, 62-84 years), and the time since their index CEA surgery ranged between 24 and 106 months. Two patients presented with signs of infection in the neck area, characterized by a draining sinus (Figure 1), and their diagnosis was made through color duplex ultrasonography. Four patients presented with the appearance of a pseudoaneurysm, and their diagnosis was confirmed using color-enhanced Duplex ultrasound and DSA (Figure 2). The seventh patient presented at the emergency vascular surgery clinic with massive bleeding following a patch rupture and unfortunately passed away before reaching the operating room. Six patients underwent surgery on a routine basis, during which the infected material was removed, and a venous patch (using the ipsi- or contralateral greater saphenous vein) was placed. The removed material was finally sent for culture analysis and the microorganisms isolated were: *Pseudomonas aeruginosa* and *Prevotella* species in one case, *Staphylococcus epidermidis* in two cases, *Pseudomonas aeruginosa *in one case, and methicillin-susceptible *Staphylococcus aureus* (MSSA) in two cases.

Outcomes

All patients remained in follow-up, with a median follow-up duration of 159 months (range, 59-201). No patients were lost to follow-up. There were no occurrences of peri-operative deaths or strokes, and no patients experienced cranial nerve injuries. Continuous clinical examinations and Duplex ultrasound checks were performed on all patients during their follow-up. Throughout this follow-up period, no patients experienced strokes or developed reinfections. It should be noted that two patients passed away, but their deaths were unrelated to strokes or reinfections. Table 1 provides an overview of the patient presentations and how their cases were managed.

**Table 1:** Presentation and management of patients with carotid patch infections OR: operation room; MSSA: methicillin susceptible *Staphylococcus aureus*

Case	Presentation	Time from index operation (months)	Management	Pathogen	Follow-up (months)	Reinfection
1	Draining sinus	26	Saphenous patch	*Pseudomonas aeruginosa*, *Prevotella* spp.	158	No
2	Draining sinus	29	Saphenous patch	Pseudomonas aeruginosa	201	No
3	Pseudoaneurysm	33	Saphenous patch	Staphylococcus epidermidis	141	No
4	Pseudoaneurysm	35	Saphenous patch	Staphylococcus epidermidis	198	No
5	Pseudoaneurysm	85	Saphenous patch	*Staphylococcus* *aureus* (MSSA)	159 (died)	No
6	Pseudoaneurysm	106	Saphenous patch	*Staphylococcus* *aureus* (MSSA)	59 (died)	No
7	Bleeding	24	Died of hypovolemic shock before getting to OR	n/a	n/a	n/a

## Discussion

Literature review

**Figure 3:**
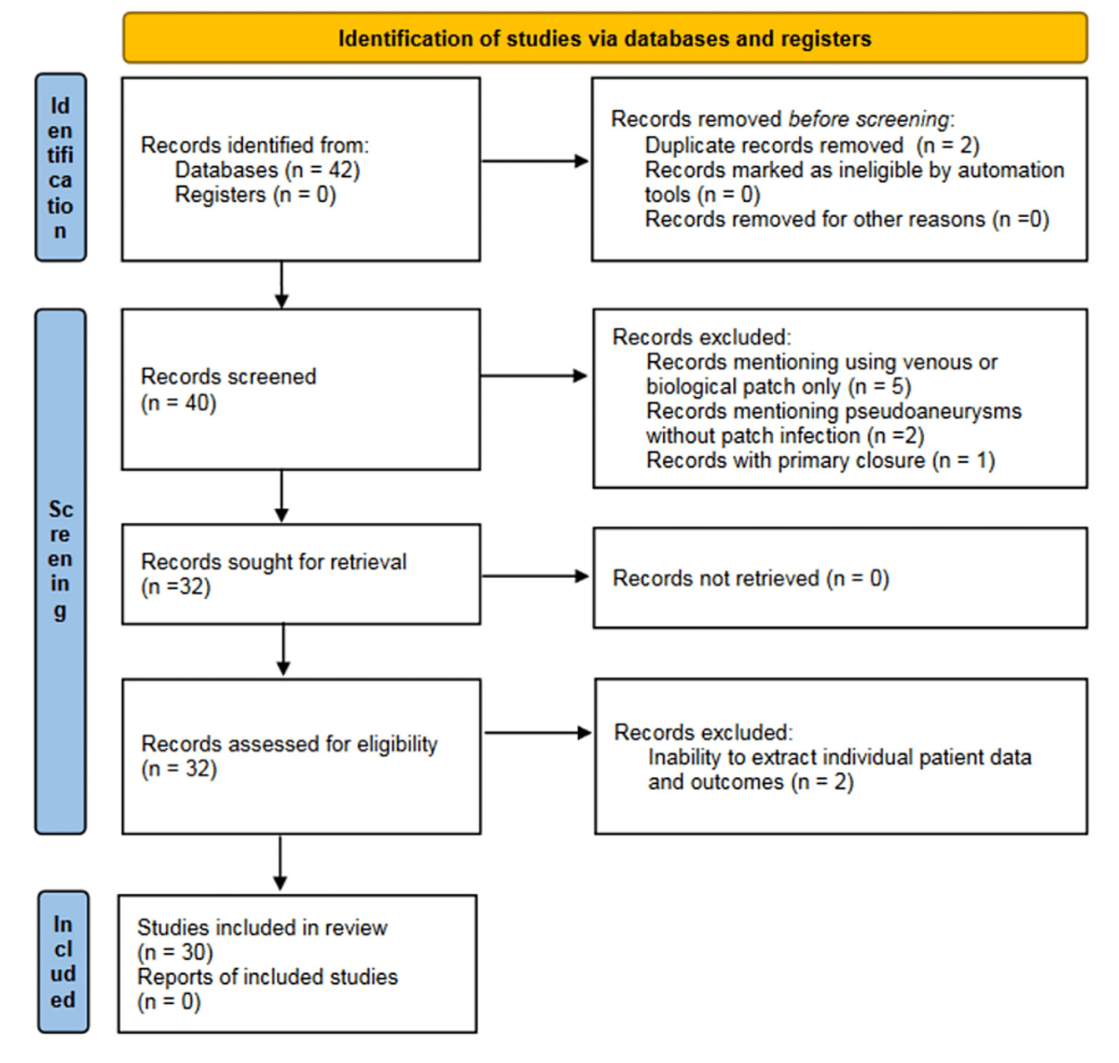
PRISMA flow diagram of the CEA patch infection studies identified and included for review PRISMA: Preferred Reporting Items for Systematic Reviews and Meta-Analyses; CEA: carotid endarterectomy

A literature review was conducted of studies of patients with prosthetic patch infection following CEA published from January 1992 to December 31, 2022. The studies were identified by a PubMed search, using the search terms “patch infection”, “synthetic patch infection”, “polyester patch infection”, “Dacron patch infection”, “PTFE patch infection”, “ePTFE patch infection”, and “carotid pseudoaneurysm”. Patients with suspected patch infection after having undergone venous patch or another biological patching (eg. bovine pericardium) were excluded from this review, as were case series of patients treated for pseudoaneurysms with no suspicion of infection or following reversed endarterectomy or primary closure. A total of 32 eligible studies were identified [2,4-35]. Two were excluded due to an inability to extract individual patient data and outcomes [34,35]. The remaining 30 series (157 patients) were included in the review [2,4-33], after double-checking for any duplicate records (Figure 3).

Extracted data was analyzed statistically in a Microsoft Excel 2021 (Microsoft Corporation, Redmond, Washington, United States) sheet. Only descriptive statistics were utilized to analyze the extracted data in this review. This included the calculation of means, medians, standard deviations, and percentages to summarize and present the data. No inferential statistical tests were performed.

The majority of included studies were either case reports or case series, which are inherently lower in the hierarchy of evidence. As such, the quality of these studies might be considered low due to their descriptive nature, lack of control groups, and potential for bias. Each study was evaluated using the Joanna Briggs Institute critical appraisal checklist for case reports and case series (2017 version), focusing on aspects such as clarity of reporting, diagnostic criteria, and follow-up.

Table 2 compiles the data extracted from published studies in the literature concerning carotid patch infections following CEA in a summarized way. A total of 157 prosthetic patch infections have been reported between 1992 and 2022 [2,4-33]. The modes of clinical presentation reported in all cases were: drainage sinus (purulent or serous) in 84 patients (53.5%), neck swelling or mass in 43 patients (27.4%), abscess in 41 patients (26.1%), pseudoaneurysm in 25 patients (15.9%) and bleeding/rupture in 16 patients (10.2%).

**Table 2:** Summary of published studies on carotid patch infections ePTFE: expanded polytetrafluoroethylene; U/S: ultrasonography; CT: computer tomography; CTA: computer tomography angiography; DSA: digital subtraction angiography; MRI: magnetic resonance imaging; PET/CT: positron emission tomography/computed tomography; SPECT/CT: single-photon emission computed tomography/computed tomography; CNS: coagulase negative *Staphylococcus*; MRSA: methicillin resistant *Staphylococcus aureus; *MRSE: methicillin resistant *Staphylococcus epidermidis*; CCA: common carotid artery; ICA: internal carotid artery; ECA: external carotid artery; CAS: carotid artery stenting; HTN: hypertension; HCL: hypercholesterolemia; CAD: coronary artery disease; CKD: chronic kidney disease; DM: diabetes mellitus; CABG: coronary artery bypass graft surgery; PAD: peripheral arterial disease; CVA: cerebrovascular accident; TIA: transient ischemic attack; CHF: congestive heart failure

Study	Number of cases	Gender (%)	Mean age, years	Age range, years	Co-morbidities	Time from index operation (mean), in days	Patch type, n (%)	Clinical manifestation, n, (%)	Pathogen, n (%)	Diagnostic modality, n (%)	Treatment modality, n (%)	Follow-up	Morbidity/ mortality
Dougherty et al. (1997) [5]	1	Female (100%)	66	n/a	HCL, smoking	180	Polyester	Purulent drainage from sinus and pseudoaneurysm	*Staphylococcus epidermidis*	Duplex U/S, DSA	Debridement, ICA-patch resection and saphenous bypass	26 months	Proximal anastomosis revision due to stenosis
Rizzo et al. (2000) [6]	8	Male (75%), female (25%)	68	48-85	HTN and/or CAD (75%), DM (62%)	274	Polyester	Draining cutaneous sinus (50%), local tenderness (25%), abscess (25%), cervical mass (12.5%), fever (12.5%), pseudoaneurysm (12.5%)	*Staphylococcus* spp (50%), *Streptococcus* spp (37.5%), None (12.5%)	Duplex U/S, DSA	Debridement (100%), patch excision and saphenous patch (62.5%), ICA-patch resection and venous interposition graft (37.5%)	3-36 months	No reinfections, 1 patient died in car accident
Byer et al. (2001) [7]	1	Male (100%)	79	n/a	HTN, CAD/CABG, progressive exertional angina, aortic stenosis, mitral insufficiency	730	Polyester	Draining sinus, mild tenderness	MRSA	CT	IV and oral antibiotics (conservative)	24 months	No reinfection, no death
Naylor et al. (2002) [8]	8	-	-	-	-	381	Polyester (87.5%), ePTFE (12.5%)	Chronic sinus (37.5%), abscess (12.5%), patch dehiscence (12.5%), massive hemorrhage (12.5%), pseudoaneurysm (12.5%), deep tissue infection (12.5%)	MRSA (37.5%), *Staphylococcus epidermidis *(25%), hemolytic *Streptococcus *(12.5%), *Staphylococcus aureus* (12.5%), *Pseudomonas* spp (12.5%)	Duplex U/S	Debridement (100%), ECA/CCA/ICA ligation (37.5%), ICA-patch resection and saphenous bypass (37.5%), patch excision and saphenous patch (12.5%), penicillin wound irrigation (12.5%)	12-96 months	No reinfections, 1 disabling CVA
Rockman et al. (2003) [9]	10	Male (30%), female (70%)	73	57-84	DM (40%), alcohol abuse (10%), steroid therapy (10%)	259	Polyester	Draining sinus (50%), cellulitis (50%), abscess (40%), hematoma (10%)	*Staphylococcus epidermidis *(40%), Streptococcus viridans (30%), Bacteroides fragilis (20%), Staphylococcus aureus (20%), Bacterium acnes (10%)	CT (60%), duplex U/S (40%), MRA (10%), DSA (10%), None (20%)	Debridement and antibiotic solution irrigation (100%), patch excision (80%), saphenous patch (60%), internal jugular patch (10%), superficial femoral vein patch (10%), ICA-patch resection and saphenous graft (20%)	8-56 months	No reinfections, no deaths
Borazjani et al. (2003) [10]	4	Male (100%)	63	52-76	HTN (50%), HCL (25%), CVA (25%), subclavian artery steal syndrome (25%), PAD (25%)	2018	Polyester	Pseudoaneurysm (100%), cutaneous sinus (50%)	*Staphylococcus epidermidis *(50%), None (50%)	Duplex U/S (75%), MRA (25%)	Debridement and pseudoaneurysm excision (100%), patch excision and saphenous patch (75%), ICA-patch resection and ePTFE interposition graft (25%)	-	No reinfections, 1 patient died on post-op day 2
Grego et al. (2003) [11]	1	-	-	-	-	42	ePTFE	Neck hematoma	*Staphylococcus aureus*	-	Hematoma and patch excision and saphenous interposition graft	-	-
Baril et al. (2004) [12]	1	Female (100%)	79	n/a	HTN, thyroid cancer and thyroidectomy, neck radiotherapy, vocal cord paralysis	1825	Polyester	Sinus	*Staphylococcus epidermidis*	MRI	CAS	12 months	No reinfection, no death
Lazaris et al. (2005) [4]	4	Female (100%)	76	74-80	-	495	Polyester	Sinus (75%), minimal wound discharge (25%)	None	Duplex U/S	Patch excision, debridement, and vein bypass	-	-
Lewis et al. (2005) [13]	1	Male (100%)	62	n/a	TIAs, contralateral carotid artery stenosis	2920	Polyester	Neck swelling, abscess and pseudoaneurysm	None	Duplex U/S, MRA	1st: oral antibiotics for 6 months, 2nd: CAS (Wallgraft™ Endoprosthesis)	12 months	No reinfection, no death
Litwinski et al. (2006) [14]	2	Male (50%), female (50%)	72	68-75	CAD/CABG (50%), mediastinitis (50%), knee septic arthritis (50%), diffuse aneurysmal disease (50%)	2095	Hemashield (50%), unknown (50%)	Neck swelling, pain and voice hoarseness (50%), pulsatile mass/pseudoaneurysm (50%)	MRSA (50%), none (50%)	Fine needle aspiration (50%), DSA (50%), duplex U/S (50%), MRA (50%)	Debridement and ICA with patch resection (100%), interposition vein graft (100%), sternocleidomastoid muscle flap (50%)	-	1 patient had a non-disabling intraoperative CVA, 1 patient had popliteal artery aneurysm rupture
Krishnan and Clowes (2006) [15]	1	Female (100%)	78	n/a	HTN, HCL	450	Polyester	Painless neck mass	CNS	Duplex U/S, CT, MRI, MRA	Debridement, subcutaneous mass excision, ICA-patch resection and saphenous bypass	-	-
Asciutto et al. (2007) [16]	6	Male (67%), female (33%)	69	48-81	DM (50%)	134	Polyester	Draining sinus (33%), abscess (33%), pain (33%), fever (33%), patch disruption (17%), pseudoaneurysm (17%)	*Staphylococcus aureus* (33%), CNS (17%), none (50%)	Duplex U/S and CT (100%)	Debridement (100%), ICA-patch resection and vein interposition graft (83%), patch excision and vein patch (17%)	44-75 months	No reinfections, 2 patients died of unrelated causes
Chaudhuri et al. (2007) [17]	1	Male (100%)	62	n/a	Ex-smoker, HCL, total laryngectomy, permanent tracheostomy & neck radiotherapy for laryngeal carcinoma, CAD/CABG	1095	Polyester	Purulent discharge from neck wound, sinus	CNS	Duplex U/S, CT	ICA balloon occlusion test, debridement, CCA/ECA/ICA-patch resection and ICA ligation	-	Transient oculomotor nerve palsy on post-op day 1, no reinfection, no death
Knight and Tait (2009) [2]	2	Male (50%), female (50%)	80	75-85	-	945	Polyester	Painless neck mass (100%), discharging sinus (50%)	*Staphylococcus epidermidis* (50%), None (50%)	Duplex U/S (100%)	Excision of infected phlegmon	8-78 months	1 patient developed a discharging sinus, no deaths
Illumninati et al. (2009) [18]	1	Male (100%)	58	n/a	-	90	Polyester	Neck mass, pain	*Staphylococcus epidermidis*	Clinical examination	Debridement, antibiotic irrigation, CCA-to-ICA ePTFE bypass	12 months	No reinfection, no death
Naughton et al. (2010) [19]	12	Male (75%), Female (25%)	78	63-84	DM (75%)	679	Polyester (59%), ePTFE (25%), saphenous (8%), bovine (8%)	Neck swelling-abscess (33%), bleeding/rupture (25%), pseudoaneurysm (25%), drainage sinus (17%)	MRSA (17%), *Staphylococcus aureus* (8%), *Streptococcus anginous* (8%), *Corynebacterium propionibacterium* (8%), none (42%), no cultures taken (17%)	Duplex U/S (75%), CT or MRA (75%), none (25%)	Debridement (100%), ICA-patch resection (92%), SFA interposition graft (50%), cryopreserved arterial allograft (25%), saphenous graft (17%), patch excision and primary arteriotomy closure (8%)	3-108 months	Hypoglossal nerve injury (1 patient), superficial skin infection (2 patients), immediately post-op neck haematoma (1 patient), no reinfections, no deaths
Harrison et al. (2010) [20]	1	Female (100%)	62	n/a	HTN	730	Polyester	Neck swelling with sinus, discomfort	None	Duplex U/S, MRI, MRA, DSA, white blood cell scan	ECA embolisation and CAS (Wallgraft™ Endoprosthesis)	32 months	Death of unrelated CAD & bronchopneumonia
Stone et al. (2011) [21]	25	-	67	47-85	DM (44%)	178	ePTFE (84%), bovine (8%), polyester (8%)	Drainage sinus purulent or serous (52%), neck swelling/abscess (20%), pseudoaneurysm (16%), bleeding (12%)	MSSA (28%), MRSE (16%), Enterobacter aerogenes (16%), Streptococcus agalactiae (8%), MRSA (8%), Proteus (4%), Serratia (4%), none (20%)	duplex U/S (56%), CT (32%), none (20%)	Incision and drainage with IV/oral antibiotics (68%), debridement (32%), patch excision and vein patch angioplasty with sternocleidomastoid flap (16%), ICA-patch resection and vein interposition graft (16%)	1-87 months	Recurrent bleed (1 patient), hoarseness (1 patient), non-disabling CVA (1 patient), CVA/death (1 patient)
Kragsterman et al. (2011) [22]	3	Female (100%)	62	16-85	-	668	Polyester	Painless mass and local fistula (100%)	CNS (33%), none (67%)	Duplex U/S, PET/CT	EndoVAC technique: stent-grafting, surgical debridement, and vacuum-assisted wound closure (VAC)	6-16 months	Transient hypoglossal nerve palsy (1 patient)
Mann et al. (2012) [23]	14	-	-	-	-	735	Polyester	Sinus (43%), false aneurysm (29%), patch rupture (7%), abscess (21%)	CNS (36%), *E.coli* (14%), MRSA (7%), *Pseudomonas* (7%), None (30%), No cultures taken (6%)	Duplex U/S	Vein bypass (72%), ICA ligation (14%), vein patch (7%), CAS (7%)	2.5-180 months	Cranial nerve injury (9 patients), CVA post-op (1 patient), death of unrelated causes (8 patients), no reinfections
Menna et al. (2014) [24]	1	Female (100%)	81	n/a	HTN, HCL, left carotid artery stenting, thyroidectomy, right quadrantectomy for in situ breast cancer	2555	Polyester	Pulsatile neck mass	*Staphylococcus epidermidis*	Duplex U/S, CT	Saphenous vein bypass, patch excision	7 months	No reinfection, no death
Thorbjørnsen et al. (2016) [25]	6	Male (67%), female (33%)	68	63-77	-	434	Polyester	Neck mass with fistula (83%), pseudoaneurysm (17%)	CNS (50%), Staphylococcus aureus (17%), Propionibacterium acnes (33%), None (33%)	-	EndoVAC hybrid treatment (relining of the infected reconstruction with a stent-graft, surgical revision of the infected area, and VAC therapy)	3-68 months	No reinfections, transient hypoglossal nerve palsy (1 patient), moderate stent-graft stenosis (1 patient), death of unrelated CAD (1 patient)
Alawy et al. (2017) [26]	8	Male (38%), female (62%)	73	49-84	DM (13%), smoking (88%), HTN (88%), CAD (50%), CKD (25%)	37	Polyester	Discharging sinus	MRSA (12.5%), None (87.5%)	Duplex U/S, CTA	Debridement and muscle flap (25%), patch removal and CCA-ICA vein bypass (12.5%), ICA ligation and CCA-ECA vein bypass (12.5%), patch removal and vein patch (37.5%), conservative with IV/oral antibiotics (12.5%)	12-48 months	No reinfections, pseudoaneurysm at 6 months post-op (1 patient), massive CVA immediately post-op/death (1 patient), death of unrelated CAD (1 patient)
Xu et al. (2017) [27]	1	Male (100%)	83	n/a	Ipsilateral TIA, contralateral internal carotid artery occlusion, bilateral vertebral artery stenoses, contralateral CVA, HTN	1460	Polyester	Sinus, pus, bleeding	*Klebsiella pneumoniae*	CTA, PET/CT, duplex U/S, SPECT/CT	1st: CAS (Fluency Plus & Viabahn stent-grafts), 2nd: surgical excision, sternocleidomastoid flap	1 month	No reinfection, no death
Fok et al. (2018) [28]	1	Male (100%)	81	n/a	Loose tooth	25	Polyester	Discharging sinus	*Streptococcus viridans*	CTA	Surgical excision & saphenous patch	3 months	No reinfection, no death
Fatima et al. (2019) [29]	29	Male (76%), female (24%)	70	61-79	HTN (93%), HCL (86%), smoking (72%), CHF (51%), CAD (48%), DM (31%), CKD (17%)	450	Polyester (31%), bovine (17%), ePTFE (10.5%), other prosthetic (10.5%), saphenous (3.5%), unknown (27.5%)	Purulent drainage or abscess (66%), neck mass (45%), erythema (41%), carotidynia or cervical pain (38%), bleeding (24%), fever (17%)	*Streptococcus* spp or *Staphylococcus* spp (51.7%), *Klebsiella oxytoca* (3.5%), *Pseudomonas aeruginosa* (3.5%), *Enterobacter aerogenes* (3.5%), *Corynebacterium* (3.5%), None (34.3%)	CTA (93%), duplex U/S (45%), MRI (17%), DSA (7%)	Surgical excision & femoral vein interposition bypass (83%), surgical excision & saphenous patch (14%), surgical excision & femoral vein patch (3%), pectoralis muscle flap (7%)	17 +/- 14 months	Transient leg swelling (1 patient), transient femoral cutaneous nerve palsy (2 patients), transient cranial nerve injury (8 patients), major cardiac event (4 patients), respiratory failure (3 patients), bleeding/revision operation (1 patient), in-hospital CVA/death (2 patients), no reinfections
Azouz et al. (2019) [30]	1	Female (100%)	91	n/a	Ipsilateral CVA, previous ipsilateral operations due to pseudoaneurysm formation after bovine pericardium patch CEA	480	Bovine	Neck mass (pseudoaneurysm), voice hoarseness	*Pseudomonas multocida*	Duplex U/S, CTA	1st: revision angioplasty (bovine patch), 2nd: surgical excision & saphenous patch, sternocleidomastoid flap	6 months	No reinfection, no death
Haddad et al. (2019) [31]	1	Male (100%)	76	n/a	Ex-smoker, CAD, bilateral CEA	4015	Polyester	Draining sinus (both operated with CEA neck sides), bleeding	*Escherichia coli*, CNS	CTA	Surgical excision & reversed saphenous interposition bypass	12 months	Transient glossopharyngeal palsy, moderate proximal anastomosis stenosis
Torres-Blanco et al. (2021) [32]	1	Male (100%)	70	n/a	Smoking, HTN, chronic myeloproliferative syndrome	2430	Polyester	Draining sinus (hemoserous ooze)	*Staphylococcus epidermidis*	Duplex U/S, CTA	Excision of sinus tract, rifampicin irrigation & preservation of the patch	24 months	No reinfection, no death
Wang and Mohan(2022) [33]	1	Male (100%)	76	n/a	-	2555	Polyester	Neck pain and swelling (pseudoaneurysm)	None	CTA	1st: CAS (Viabahn stent-graft & ECA coiling), 2nd: surgical excision, 3 weeks of VAC therapy	3 weeks	No reinfection, no death

Numerous microorganisms have been isolated on cultures of excised infected material [2,5,10-22]: *Staphylococcus epidermidis* (n=22; 14%), *Staphylococcus aureus* (n=15; 9.6%), Coagulase-negative *Staphylococcus* (n=13; 8.3%), MRSA (n=12; 7.6%), *Streptococcus viridans* (n=8; 5.1%), *Enterobacter aerogenes* (n=6; 3.8%), *Pseudomonas* spp. (n=5; 3.2%), *Bacteroides acnes* (n=3; 1.9%), *Corynebacterium* spp. (n=3; 1.9%), *Klebsiella* spp. (n=3; 1.9%), *Escerichia coli* (n=3; 1.9%), *Streptococcus agalactiae* (n=2; 1.3%), *Streptococcus anginous* (n=1; 0.6%), *Streptococcus mitis* (n=1; 0.6%), β-hemolytic *Streptococcus* (1; 0.6%), *Bacteroides fragilis* (2; 1.3%), *Proteus *spp. (1; 0.6%), and *Serratia* spp. (1; 0.6%). In many cases, two microorganisms have been isolated [5,10]. Perioperative antibiotic therapy might be the cause of negative culture in many cases (n=50; 31.8%).

Treatment was as follows in these studies: vein graft in 65 patients (41.4%), vein patch in 33 patients (21%), only incision and drainage in 17 patients (10.8%), only antibiotics irrigation in 13 patients (8.3%), arterial graft in nine patients (5.7%), excision of infected material, antibiotic irrigation, and sternocleidomastoid flaps in six patients (7.2%), excision of infected material and ICA ligation in seven patients (4.5%), cadaveric homograft in three patients (3.6%), and patch removal and primary arterial closure in one patient (0.6%).

According to Stone et al., who presented a total of 25 cases, treatment choice was affected by the type of infection, as well as by the surgeon's experience [21]. Overall, 17 patients (68%) underwent incision and drainage with antibiotics, and eight patients (32%) underwent definitive surgical treatment; four (50%) received patch excision with vein patch angioplasty and sternocleidomastoid flaps and four (50%) received patch excision with vein graft interposition. Of the 17 patients on patch preservation, 13 (76.5%) were managed by cardiothoracic or general surgeons rather than a vascular surgeon, whereas all eight cases with patch excision and vein patch angioplasty or vein interposition grafting (100%) were performed by a vascular surgeon.

Finally, the series by Chiesa et al. [34] and Sabrout et al. [35], although reporting 21 and 13 cases, respectively, were not included in our review due to a lack of data regarding the description of the presentation and any pathogens identified by cultures taken (See also Figure 3).

Prosthetic patch infection following CEA is a relatively infrequent but also underreported complication. The literature indicates a low infection rate of synthetic patches, ranging from 0.25% to 1% [2,9,34-38], despite the limited number of reported cases worldwide: only 157 so far. Pseudoaneurysms after CEA occur at an overall rate of 1%, with infected pseudoaneurysms accounting for 0.025-0.625% of cases [36-38].

The timing of symptom appearance shows significant variation, ranging from a few days to several years after the index surgery. It appears to follow a two-peaked distribution pattern, with about one-third of cases manifesting within two months of the initial procedure and the remaining two-thirds presenting later, often with persistent sinuses or pseudoaneurysms. *Staphylococcus* and *Streptococcus* are the predominant pathogens in these instances, emphasizing the need for timely administration of appropriate antimicrobial therapy until culture results become available. The precise factors influencing this timeline are not fully understood, although the type of pathogen and the material used for patching seem to have significant influences. Some medical facilities have opted to exclusively utilize bovine pericardial patches, perceiving them as more resistant to infections [23].

The identification of patch infection is generally uncomplicated in most cases. The emergence of symptoms like sinus drainage, neck swelling, abscess, pseudoaneurysm, bleeding, or rupture, along with heightened inflammatory markers in patients with a history of CEA, should prompt immediate suspicion. Typically, confirming the diagnosis involves various imaging techniques such as ultrasonography, DSA, MRI/MRA, CT/CTA [2,21,39]. Duplex ultrasound is commonly the initial investigative procedure employed to detect patch infections. It enables the visualization of patch irregularities, which can act as an early indicator of potential infection, in addition to the examination of deep-seated collections and pseudoaneurysms. According to the 2020 European Society for Vascular Surgery (ESVS) clinical practice guidelines on the management of vascular graft and endograft infections, for suspected vascular graft/endograft infection, CTA is recommended as the first line diagnostic modality, and the use of ultrasound as the sole diagnostic modality is not recommended. For patients suspected of vascular graft/endograft infection, if CTA is contra-indicated, the use of MRA may be considered, whereas in cases of non-convincing findings on CTA, the use of 18-fluoro-deoxyglucose positron emission tomography (18F-FDG-PET) combined with low dose CT or single PET-CT, if available, are recommended as additional imaging modalities to improve diagnostic accuracy. Cross-sectional imaging also assists in the assessment of the upper ICA and aids in planning potential access to the skull base [23].

Managing patch infections can be challenging and should be undertaken by experienced centers due to the complex anatomy of the region and the potential for serious complications like stroke and cranial nerve injury. The primary goal of treatment is to remove the infected material, followed by revascularization. Surgical exploration, debridement of infected tissue, and patch excision, combined with revascularization, are considered the standard management strategy. Options for autologous reconstruction include vein patch closure, vein bypass, or a bypass using an autologous arterial conduit, such as the superficial femoral artery. In instances of MRSA infection, where carotid ligation might be chosen, although traditionally linked to a heightened risk of perioperative stroke, its necessity should be evaluated, considering preoperative examinations of collateral circulation and the patient's tolerance levels [23]. The use of a sternocleidomastoid muscle flap has been shown experimentally and clinically to improve healing time, lower bacterial counts, and bring well-vascularized tissue to a clinically infected area, such as the infected surgical wound post-CEA [40].

A novel hybrid procedure, the EndoVAC technique, consisting of relining of the infected reconstruction with a stent graft, surgical revision, and vacuum-assisted closure (VAC) therapy to permit granulation and secondary/delayed healing has been used by some clinicians with promising results. This technique offers itself as an alternative, less invasive, option for the treatment of infected vascular reconstructions in selected cases, when neither traditional radical surgery, nor conservative simple negative pressure wound therapy is considered feasible or safe [25].

Finally, a small subgroup of patients has been reported to have undergone treatment with covered stents, showing promising perioperative outcomes [12,13,20,22,23,25,27,33]. However, long-term data regarding patency and late reinfection are currently insufficient. It is important to note that literature on the role of covered stents may be biased, as centers tend to publish favorable outcomes.

Limitations and future directions

Our study is limited by the small number of patients and the fact that it is a single-center study. The review is limited by the low methodological quality of the included studies, many of them being case series, which may affect the generalizability and robustness of our findings. The rarity of CEA patch infection makes the design of large-scale studies challenging, further constraining the available evidence. Nonetheless, our results underscore the need for future research, including experimental studies and the development of novel techniques, to better understand and manage this clinical issue. Advancements in diagnostic tools and innovative treatment approaches could provide valuable insights and improve patient outcomes in the context of CEA patch infection.

## Conclusions

Patch infection following carotid endarterectomy is relatively rare. The primary culprits behind these infections are often *Staphylococcus epidermidis* and *Staphylococcus aureus*. Clinical presentations may include drainage sinus (purulent or serous), neck swelling with abscess formation, pseudoaneurysm development, and bleeding/rupture. The prevailing treatment approach typically involves excising the infected material, followed by revascularization using a vein patch or graft. Maintaining a strong emphasis on preventive measures remains of paramount importance in real-world clinical practice to avoid mortality and morbidity from this uncommon but serious post-CEA complication.
